# A Medium-Term Field Experiment to Study the Effect of Managing Soil Chemical Properties on Fusarium Wilt in Banana (*Musa* AAA)

**DOI:** 10.3390/jof7040261

**Published:** 2021-03-31

**Authors:** Rafael A. Segura M., Jetse J. Stoorvogel, Fabio A. Blanco R., Jorge A. Sandoval F.

**Affiliations:** 1 Soil Geography and Landscape Group, Wageningen University, 6708 PB Wageningen, The Netherlands; jetse.stoorvogel@wur.nl; 2 Research Center, CORBANA S.A., Guápiles 32-7210, Costa Rica; jsandoval@corbana.co.cr; 3 Independent Consultant, Barva 40202, Costa Rica; fabioa.blanco@gmail.com

**Keywords:** Costa Rica, Panama disease, plant nutrition, soil fertility, soil type

## Abstract

*Fusarium oxysporum* f. sp. *cubense* (Foc) is a soil-borne fungus causing Fusarium wilt (FW) in banana. It is practically impossible to eradicate Foc in soils. Our understanding of soil–Foc–banana interactions is hampered by inconsistent research results caused by agro-ecological variability and the complexity of the soil system. This study aimed to evaluate the options to manage soil chemical properties to reduce disease expression and maintain banana production. The expression of FW (Foc Race 1) and the agronomic performance of the Gros Michel (*Musa* AAA) banana were evaluated in two medium-term factorial field experiments at representative locations in the Costa Rican banana region. In the experiments, five soil chemical properties (pH, N, Ca, Mg, and Mn) were managed to achieve a low and a high level. Plant mortality caused by FW, soil fertility, plant nutrition, and agronomic performance were monitored during four crop cycles. After the first crop cycle, the treatments started to present differences in plant mortality. There was a significant rise of plant mortality after the second crop cycle resulting in a cumulative plant mortality exceeding 60% in both experiments. A lower soil pH consistently resulted in significantly higher plant mortality. The interactions between soil properties (pH-N, pH-CaMg, pH-Mn, N-Mn, and CaMg-Mn) also influenced plant mortality. Soil N was the most significant treatment affecting leaf nutrient concentrations, bunch weight, and clusters per bunch. The experiments confirmed the potential role of soil management in FW expression in banana. Our results suggest that the management of soil chemical properties in the conditions here studied may help to reduce the expression rate of FW, but not to control the disease in the long run.

## 1. Introduction

Global crop production is seriously hampered by crop diseases [1,2,3]. Fusarium wilt (FW) in banana is a prime example. FW (also known as Panama disease) is caused by the soil-borne fungus *Fusarium oxysporum* f. sp. *cubense* (Foc). Global banana production has been threatened by four Foc races: Race 1, Race 2, Subtropical Race 4, and Tropical Race 4 (TR4) [4,5]. Foc Race 1 decimated the commercial production of the Gros Michel banana (a sub-group of *Musa* AAA), which used to be the main cultivar in Latin America and the Caribbean (LAC) until a major outbreak of FW in the first half of the 20th century [6]. The social and economic impacts in the region were substantial [7]. After struggling with the disease, Cavendish (another sub-group of *Musa* AAA) was found to be naturally resistant to Foc Race 1. The most effective option to maintain banana exports was to replace the Gros Michel banana by Cavendish bananas [4,8,9,10]. Despite the availability of the resistant Cavendish, Gros Michel is still being produced in LAC in smallholder systems, as its specific traits are preferred for local trade and consumption [11]. Bananas (*Musa* AAA) that are susceptible to Race 1 (including Gros Michel) and also plantains (*Musa* ABB) remain important sources of incomes and represent around 30% of the global banana production [12,13]. At the end of the 20th century TR4 appeared in Asia and started to spread affecting most banana cultivars including the important Cavendish cultivar.

Efficient practices to eradicate or control Foc in infested soils are lacking. Foc is a highly competitive fungus and it can survive in the soil for decades [14]. Soil flooding was widely implemented to eradicate the soil-borne fungus during the outbreak of Race 1. Rather than eradicating Foc, this practice contributed to the further dissemination of Race 1 [4,15,16]. Other control practices, such as the application of fungicides, replanting, and the eradication of infested plants, were tested but with mostly negative results. In most cases, the aggressive character of the disease did not allow for further commercial banana production [4,17].

Despite a general awareness of the potential of soil management in the control of this disease [4,17,18,19,20,21,22], it is still not considered as a viable strategy to control FW in banana. The experimental results in the literature are inconsistent making it difficult to identify recommendations for soil management to control or alleviate the effect of the disease. In general, soil properties are known to influence the predisposition of crops to diseases [23,24,25]. Therefore, it is likely that crop predisposition to diseases can be influenced by soil management [26,27]. However, given the large number of soil properties, it is important to identify which soil properties influence the predisposition of a crop to diseases. Soil properties that can be changed easily through management (e.g., fertilization, liming, tillage, and drainage) can be considered as first candidates for crop disease management.

Although many soil properties and their interactions are involved in crop development and production, some soil properties are commonly referred to in the scientific literature as important drivers for disease development in crops. Nonetheless, the scientific community still lacks a proper understanding of the role of soil properties on crop disease development. In this study, we focus on soil chemical properties that can be influenced by soil management. Soil pH and nitrogen (N), for instance, are two important soil properties in crop production. Moreover, they are frequently mentioned to influence the crop response to diseases [28,29,30,31]. Other soil properties as calcium (Ca) [25,32,33], magnesium (Mg) [34], and manganese (Mn) [35] are reported to influence crop diseases [36].

The relation between soil properties and FW is mostly studied in greenhouse experiments. The advantage of the greenhouse experimentation is the possibility to vary one or a limited number of properties under controlled conditions to derive a specific relationship. The disadvantages of the greenhouse experiments with large perennial crops like banana is that it is very difficult to carry out the experiment for an entire crop cycle, let alone over multiple crop cycles. Many greenhouse experiments with banana are carried for a relatively short period of 8–15 weeks on small plants [37]. Therefore, it is very important to confirm the results from greenhouse experiments under field conditions [37]. Alternatively, farm surveys are carried out where FW is observed on farms and correlated to soil analyses from those locations [20,21]. Agro-ecological conditions can be extremely variable and require many observations to carry out multivariate analysis to unravel the complex relationships [38]. Performing field experiments in representative banana producing areas, over multiple crop cycles can provide useful information about the Foc-soil-banana interactions. Field experiments looking at the banana response to the Foc infestation over various crop cycles under natural ecological conditions can show the true potential of soil management on the disease expression and how it affects crop production.

Given the impact of FW on banana production and the ineffectiveness of current management strategies to control or eradicate the fungus from infested soils, there is an urgent need for new approaches. Genetic resistance is considered an effective solution against FW and has proven itself in the past [39,40]. However, it requires time to develop resistant varieties that are accepted by the producer and consumer. Integral solutions to face current problems caused by FW in the diverse banana scenarios are required. This paper hypothesizes on the basis of experimental proof from greenhouse experiments that soil chemical properties influence the predisposition of banana to Foc Race 1. This study aims to evaluate whether the crop behaves similarly under field conditions in Costa Rica and over multiple crop cycles in order to develop strategies to maintain the production even in Foc infestation conditions. Experiments with Gros Michel banana and Foc Race 1 may also help in preparing for the eventual arrival of Foc TR4 and the Cavendish production. The role of soil properties on Foc Race 1 was found to be similar to the role of soil properties on Foc TR4 [41]. The latter is especially relevant with the recent arrival of Foc TR4 in LAC [42,43].

## 2. Materials and Methods

### 2.1. Study Area

The main banana region of Costa Rica is located in the Caribbean lowlands in the North East of the country [44]. The region exhibits diverse soil conditions and is typically divided into two sub-regions defined by the location with respect to the Reventazón river. Soils east of the Reventazón river are predominantly sedimentary, fertile, (loamy) clay soils. Soils at the western side originate from sedimentary materials and volcanic ashes with (sandy) loam textures and an intermediate soil fertility [45,46,47]. More than 95% of the Costa Rican banana production takes place on those soil types [44].

Experimental sites were established on two locations within the representative soils used for banana production. Both experiments are located on research stations of the Costa Rican Corporation of Banana Producers (CORBANA S.A.). The experiment in the western region (Exp-west) was located at the La Rita experimental station (132 m.a.s.l.; 10°15′54″ N, 83°46′26″ W). The experiment in the eastern region (Exp-east) was located in 28 Millas (4 m.a.s.l.; 10°06′40″ N, 83°22′53″ W). Both locations have a perhumid tropical climate with an average annual rainfall of 3000–3500 mm well distributed throughout the year and with minimum temperatures around 17 °C and maximum temperatures around 35 °C.

### 2.2. Experimental Setup

**Figure 1:**
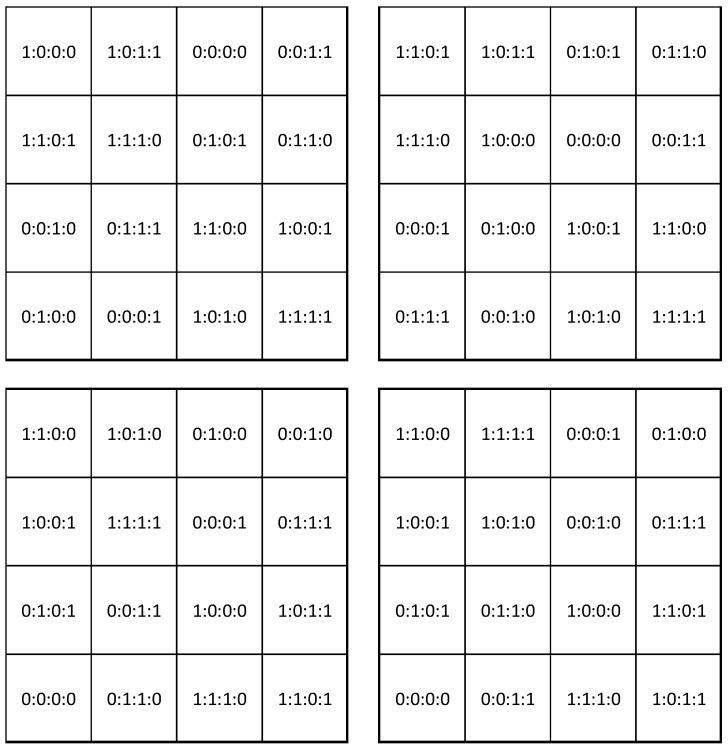
Design of the factorial experiment with 2 levels of four soil properties: pH, N, CaMg, and Mn (represented as pH:N:CaMg:Mn with a 0 for the low level and a 1 for the high level) with 4 replications of each treatment.

Exp-west covered 0.5 ha in an area that was used for banana production but fallow two years prior to the experiment. Exp-east covered 0.8 ha in an area that was used for plantain production, but that also had been fallow two years prior to the experiment. The experiments took place between November 2014 and July 2018. The fallow vegetation was cleared manually, and the residues left on the soil as mulch. Both areas had an intensive drainage infrastructure typical for Costa Rican banana plantations. The drainage channels were cleaned before the start of the experiment. The experiments were designed as a factorial trial studying the effect of four different soil properties: pH, Nitrogen (N), Calcium + Magnesium (CaMg), and Manganese (Mn). Ca and Mg are found to be highly correlated in Costa Rican banana soils [48] and are therefore included in a combined treatment in the experimental setup. Each of the soil properties were studied at two levels and the overall experiment had four replications. The area was sub-divided into 64 plots (16 treatments with 4 replications) as displayed in Figure 1. Planting was done with tissue culture Gros Michel banana plants, following standard commercial practices. In both experiments planting was done in a triangular planting pattern at a density of approx. 1600 plants per ha corresponding to an average commercial plant density. Each plot in Exp-west included 7–8 plants (486 plants in the entire experiment), whereas plots in the larger Exp-east included 15–18 plants (1160 plants in the entire experiment). The experiment was maintained for four crop cycles.

### 2.3. Plant Inoculation

Foc Race 1 is regarded as endemic in Costa Rican banana soils although the levels do vary considerably. As a result, a treatment without Foc Race 1 in the experiment would have been difficult to achieve. To ensure a homogeneous (relatively high) infestation throughout the experiments, each plant within the experiments was artificially inoculated twice with 5 g of rice colonized with Foc Race 1 collected from Costa Rican soils and cultivated by the Laboratory of Biological Control (CORBANA’s Research Center). The procedure of rice inoculation is novel and in process of publication, but the density of rice colonization was at least 1 × 10^5^ Foc conidia per g rice. The first artificial inoculation took place seven weeks after planting and a second artificial inoculation took place at the end of the 1st cycle, around 50 weeks after planting. The rice (with Foc Race 1) was buried 15 cm deep at the base of the plant for the first inoculation, and in front of the following sucker for the second inoculation. A control without inoculation was not included due to the natural soil infestation with Foc Race 1 in both experimental areas. 

### 2.4. Soil Management

Soil management is described in Table 1. The experiments were established in a complete factorial design considering four main soil chemical properties for banana production (pH, N, CaMg and Mn) with two contrasting levels (low and high) each. The experiments started with a uniform management including the application of compost at planting and a basic startup fertilization during the first 7 weeks. This startup fertilization program is standard for new plantations. Differentiation in the fertilization with N, CaMg, and Mn started in week 8. The various treatments are supplemented with a standard nutritional package for all the nutrients that were not part of the treatments. The application of this standard nutritional package also started in week 8. Changes in pH levels (either through acidification or liming) were made at three distinct moments during the experiment: before planting, at the start of the various treatments, and between cycle 2 and 3.

### 2.5. Plant Monitoring

Plant mortality was recorded as a percentage during the four crop cycles as the main indicator of the Fusarium wilt incidence. Plant mortality by FW was obtained by registering the harvested plants at the end of each crop cycle against the total plants at the beginning of the experimental period. An infested plant (showing symptoms) died in most of the cases. Plants that were not harvested almost exclusively died as a result of FW as indicated by the typical symptoms of wilting and pseudostem splitting. Those plants were not replaced during the experiment. Mortality was therefore a cumulative number over the entire experiment (four crop cycles). 

Soil chemical properties were analyzed at the beginning of the experiments [49] (before the application of any treatment), a second analysis took place between the 1st and 2nd cycle (before the third pH (liming or acidification) treatment) and a third analysis took place between the 3rd and the 4th cycle. Soils were sampled at 0–30 cm depth in front of the following sucker at flowering with a gouge auger.

Leaf samples from the medium section of the third leave of plants were taken for a complete analysis of leaf nutrient concentrations at flowering in the 1st and 2nd crop cycle. The leaf analysis included N, P, K, Ca, Mg, S, Fe, Cu, Zn, Mn and B. Agronomic performance was measured in terms of plant height, pseudostem circumference (at 1 m above the ground), and clusters or hands per bunch at flowering [44]. The bunch weight was measured at harvest (at the end of each crop cycle) 80 days after flowering (the average of the standard harvesting age in Costa Rican plantations). With the bunch weight, the density per ha and the plant mortality, a projection of the total harvested fruit ha^−1^ crop cycle^−1^ was calculated in each experiment. Sampling and data collection were carried out during the 1st and 2nd crop cycle. Due the detrimental effect of the disease on the plants after the 2nd crop cycle, the evaluation of agronomic performance and leaf nutrient analysis were not performed for the 3rd and the 4th crop cycle.

### 2.6. Statistical Analysis

Data analysis was done separately for each region but following the same protocol. The mean was modeled by a full factorial design of five fixed factors, namely pH, N, CaMg, Mn, and crop cycle. The whole data analysis was performed using R statistics [50]. 

Plant mortality was analyzed using the logit of dead plants (logit(p) = log(p/(1 − p), where p = proportion of dead plants) as response variable. The generalized estimation equation (GEE) technique was used, taking as repeated measures the counts of dead plants by FW across the 2nd, 3rd, and 4th crop cycles. The 1st crop cycle was omitted in the analysis because its plant mortality ranged within the normal limits of healthy plantations (around 2%). The binomial variance function was applied to model residues distribution since mortality outcomes are of the binomial type. Also, the first order autoregressive (ar1) correlation structure was chosen to model the repeated measures after being compared to the “independence” and “exchangeable” structures. The geeglm function of the geepack package [51,52,53]. Apart from the above-described analysis, an ordinary regression analysis of percent of plant mortality on soil pH values of the whole plots in each experiment was performed.

All the agronomic variables (plant height, pseudostem circumference, clusters or hands per bunch, and bunch weight) and the leaf nutrient concentrations were analyzed using a linear mixed model in which replicates were the only random factor and crop cycle was a repeated measures factor. Heteteroscedasticity across crop cycles and correlated measurements were accounted for through the varFixed correlation structure. The lme function of the nlme package was used for computations. The analyses of soil and leaf nutrient concentration were similar to those of the agronomic variables, except that it was not necessary to include heteroscedasticity in the model. In all the analyses, the means of main factors levels and of combinations of them were obtained and tested using the emmeans package [54].

## 3. Results

### 3.1. Plant Mortality

**Figure 2:**
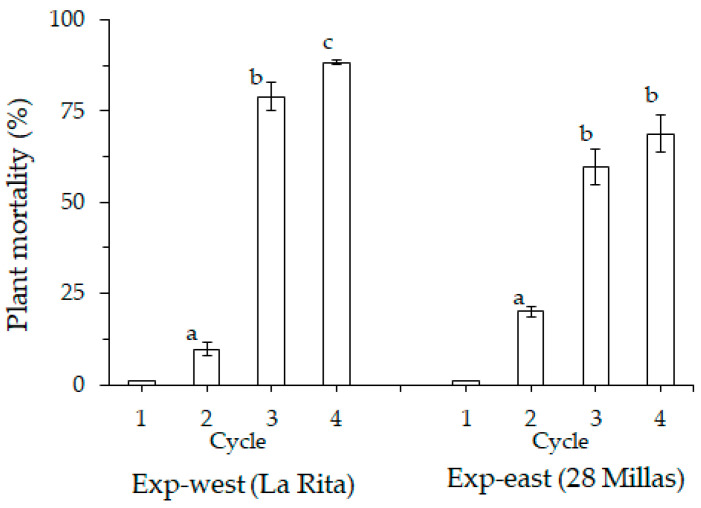
Aggregated plant mortality over all treatments by Fusarium wilt (Foc race 1) of Gros Michel banana during four crop cycles in two experiments (Exp-west in La Rita and Exp-east in 28 Millas) in the Costa Rican banana region. Bars with different letter in cycles are significantly different. *N* = 486 in Exp-west and 1646 in Exp-east. (Ierror bars represent the standard error).

Plant mortality was relatively low in the 1st and the 2nd crop cycle. However, after the second crop cycle plant mortality increased rapidly (Figure 2). In the 1st crop cycle, the average mortality was minimal (around 1%) in both experiments. The 2nd crop cycle showed an increase in mortality in the experiments, up to 11% in Exp-west and 20% in Exp-east. A rapid and significant increase (*p* ≤ 0.001) of plant mortality occurred after the 2nd crop cycle in both experiments (Figure 2).

**Figure 3:**
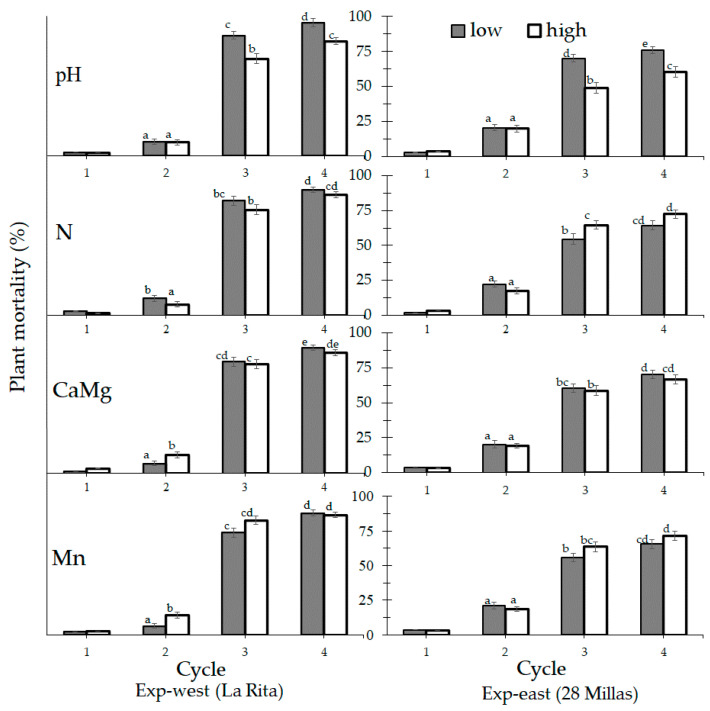
Plant mortality by Fusarium wilt (Foc race 1) of Gros Michel banana plants according to the management of four soil chemical properties (pH, N, CaMg and Mn) during four crop cycles in two experiments (Exp-west in La Rita and Exp-east in 28 Millas) within the Costa Rican banana region. Bars with different letter in cycle are significantly different according to the soil property. Total plants per treatment is 243 in Exp-west and 823 in Exp-east. (error bars represent the standard error).

Plant mortality also differed as a result of the different treatments in the 2nd, 3rd, and 4th crop cycle (Figure 3). pH-low was linked with a higher plant mortality by FW for both experiments in the 3rd (*p* ≤ 0.001) and the 4th (*p* ≤ 0.001) crop cycle. In the statistical tests, all plots are included to have a sufficient number of observations, although this may have been a source of variation in the groups. Differences in mortality between pH-low and pH-high varied from 14% to 17% in the Exp-west and from 16% to 22% in Exp-east. The N level did not influence the plant mortality in the 3rd (*p* = 0.873) and the 4th (*p* = 0.162) crop cycle in Exp-west. In Exp-east a higher plant mortality was linked with N-high only in the 3rd (*p* ≤ 0.030) crop cycle. The effect was less significant (*p* ≤ 0.070) in the 4th crop cycle, but the same trend was observed. The effect of CaMg (*p* ≤ 0.025) and Mn (*p* ≤ 0.005) in the plant mortality were significant only in the 2nd crop cycle of Exp-west. 

**Figure 4:**
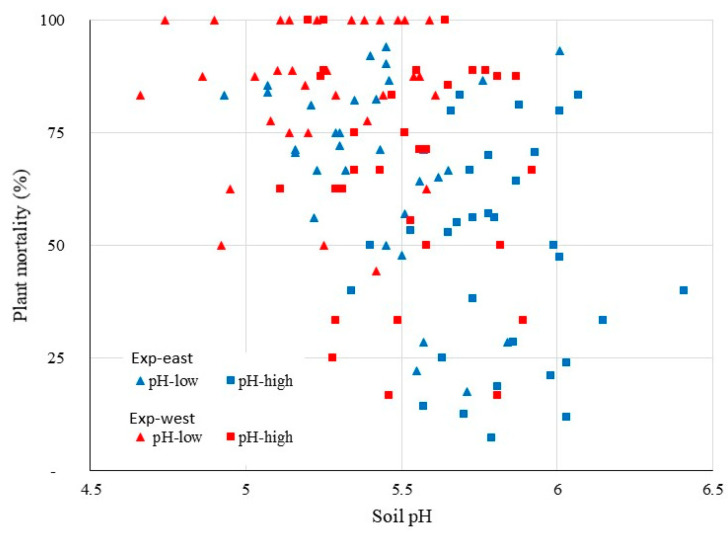
Plant mortality by Fusarium wilt (Foc Race 1) of Gros Michel banana as a function of soil pH during the 3rd and 4thcrop cycles of two experiments (Exp-west in La Rita and Exp-east in 28 Millas) within the Costa Rican banana region.

**Figure 5:**
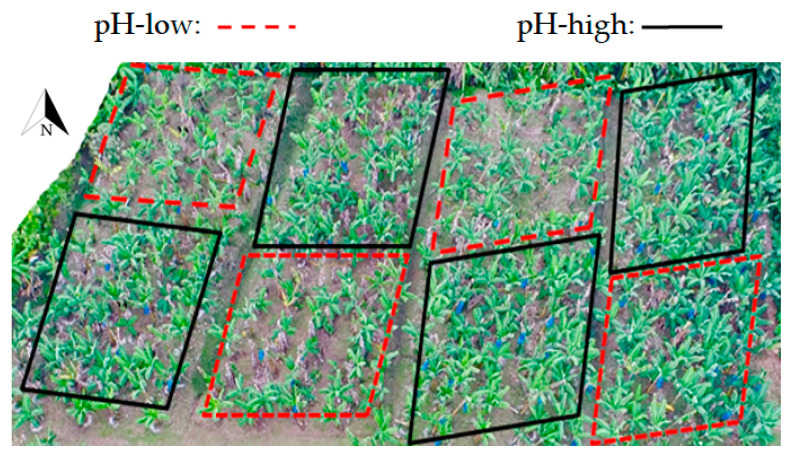
Aerial perspective from 30 m altitude of the impact of soil pH on plant mortality by Fusarium wilt (Foc race 1) of Gros Michel banana in the Exp-east in 28 Millas (Costa Rica) between the 2nd and 3rd crop cycles (aerial photo: Jean-François Michaud, CBC, Radio-Canada).

The relation between plant mortality and soil pH for each individual plot in the 3rd crop cycle for both experiments is presented in Figure 4. The inconsistent results in the literature are not surprising, if we see the scatter in the relation between pH and mortality in the figure. However, the figure clearly shows that a low soil pH always corresponds to a high plant mortality irrespective of the other conditions. The clear role of soil pH is visually confirmed on an aerial photo of the plantation between the 2nd and the 3rd crop cycle in Exp-east (Figure 5). The aerial perspective shows a clear reduction in soil cover in the pH-low plots as it was observed in both experiments.

**Figure 6:**
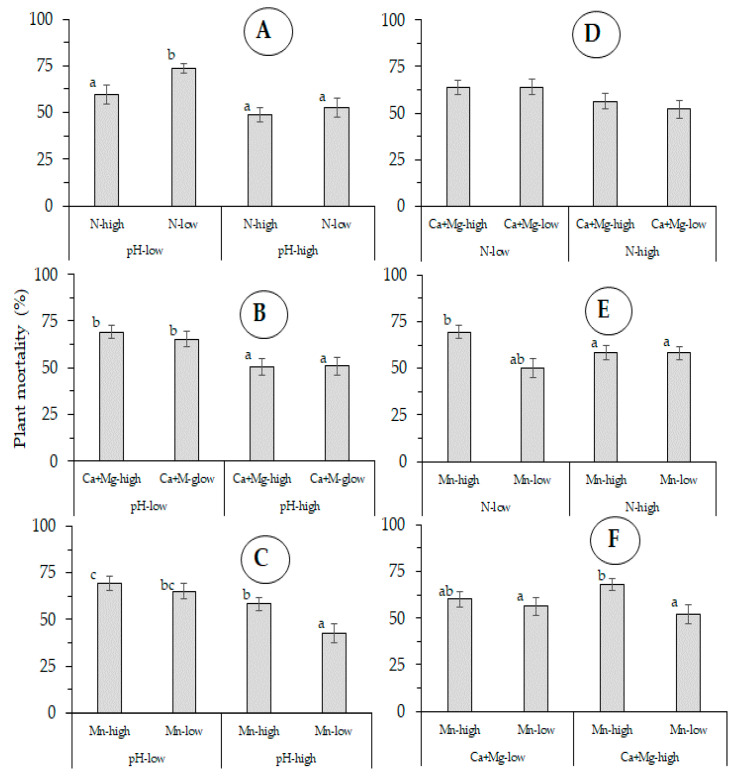
Aggregated effect of the interaction of five soil chemical properties: (**A**): pH- N, (**B**): pH-CaMg, (**C**): pH-Mn, (**D**): N-CaMg, (**E**): N-Mn and (**F**): CaMg-Mn, on plant mortality by Fusarium wilt (Foc race 1) of Gros Michel banana after four crop cycles Exp-west (La Rita, Costa Rica). (error bars represent the standard error).

**Figure 7:**
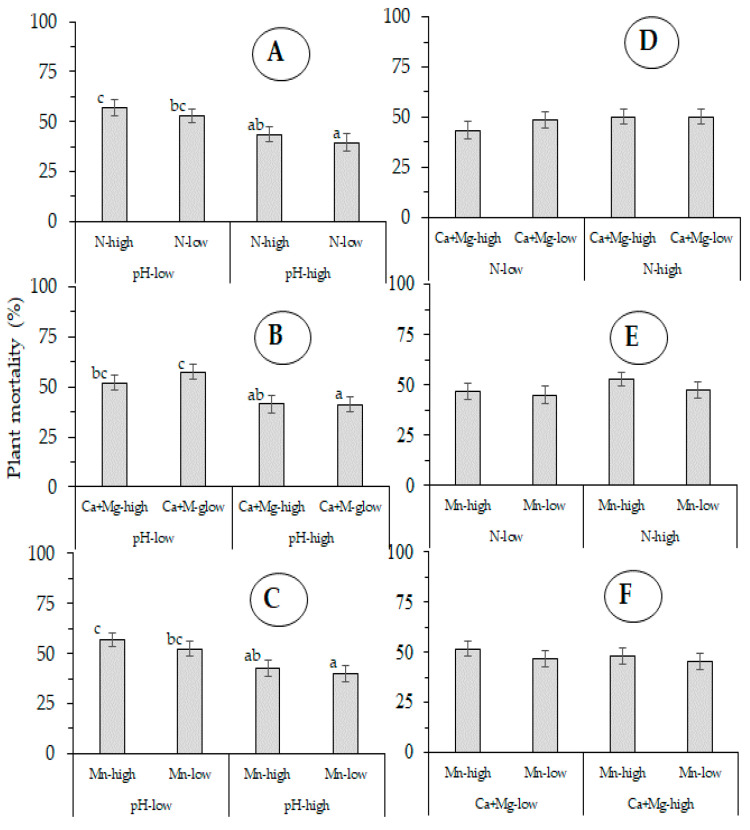
Aggregated effect of the interaction of five soil chemical properties: (**A**): pH-N, (**B**): pH-CaMg, (**C**): pH-Mn, (**D**): N-CaMg, (**E**): N-Mn and (**F**): CaMg-Mn, on plant mortality by Fusarium wilt (Foc Race 1) of Gros Michel banana after four crop cycles in Exp-east (28 Millas, Costa Rica). (error bars represent the standard error).

In Exp-west, plant mortality was also influenced by the interaction of pH-low and N-high (*p* ≤ 0.010), pH-high and Mn-high (*p* ≤ 0.016), and CaMg-low and Mn-high (*p* ≤ 0.007) (Figure 6). There were no differences in plant mortality in relation of the interaction of the tested soil chemical properties in Exp-east (Figure 7).

### 3.2. Analysis for Managed Soil Chemical Properties

Soil management resulted in significant differences in soil chemical properties in the second and the third analysis, during the four evaluated crop cycles in Exp-west. The trend in values for evaluated soil properties followed a significant trend with respect to the level for pH, Mg, and Mn. In Exp-east, only pH and Mn maintained significant differences in the second analysis. In the third analysis, only soil pH presented a significant difference according to the soil treatment. The rest of the soil chemical properties did not present significant differences under the various treatments (Table 2).

### 3.3. Leaf Nutrient Concentration

Leaf analyses only took place in the first two crop cycles. In the 3rd and 4th crop cycle plant mortality basically did not allow for proper sampling. The results of the leaf analyses are presented in Table 3. In the case of N and Mn, the concentrations of the nutrients were significantly higher with the high levels in the treatment. The case of N is very specific as almost all nutrient concentrations are significantly higher in the N-high treatment compared to the N-low treatment. The K concentrations were also found to be significantly different due to the N-treatment, but the K contents were significantly lower in the N-high treatments. Although the treatments were designed to be rather extreme, only N-treatment appeared to have an overall effect on nutrient uptake in both experiments.

### 3.4. Agronomic Performance

Managing soil chemical properties had minimal influence on agronomic performance in the 1st and the 2nd crop cycle. Only N influenced a direct effect on the bunch weight (18.8 Kg in N-low vs 19.9 kg in N-high) and clusters per bunch (8.9 in in N-low vs 9.0 in N-high) in the 1st crop cycle of the Exp-east and CaMg in the bunch weight (26.2 kg in CaMg-low vs 24.8 kg in CaMg-high) in the 1st crop cycle of Exp-west. No differences in the rest of the agronomic variables were found in the two evaluated crop cycles. There were significant (*p* ≤ 0.050) differences in the average of the agronomic variables between the cycles in both experiments. The 2nd crop cycle showed higher values respect to the 1st crop cycle, as is usual in new banana plantations (Table 4).

## 4. Discussion

### 4.1. Plant Mortality

Plant mortality in relation to soil management was analyzed during four crop cycles. Despite the significative results with soil pH and its interactions with the other tested soil properties (the nutrients in this case), the very high plant mortality after the 2nd crop cycle (exceeding the 60%) prevented to verify the effect of those nutrients on FW expression. The plant mortality observed in both experiments after the 4th crop cycle exceeded that reported in the literature [17], possibly due to the artificial inoculation method used in our study. However, the time from the first inoculation to the end of the 2nd crop cycle was probably not long enough to present a higher level of infection.

Soil pH is described in the literature as an important indicator for soil health and banana production [44,55]. Our results clearly confirm that it also plays an important role in the expression of FW in banana as it was also reported in greenhouses studies [41]. In the 3rd and the 4th crop cycle, where the soil treatments showed significant effects in plant mortality, pH maintained a consistent behavior in its influence on the disease. Relatively low mortality rates were always linked to a higher soil pH. It appears that increasing soil pH and avoiding soil acidification can reduce the impact of the disease in the medium-term.

A higher soil pH (near and up to 6.0) can be linked with improved plant development but also to the lower plant mortality caused by FW. Both direct and indirect effects of soil pH can be playing a role in the findings. As a main soil property, pH affects other biotic and abiotic soil properties [31,55,56,57]. The exact causal-effect of the suppressive effect of a higher soil pH values cannot be determined based on the current study. The variation in the effect could be caused by interactions with the other soil properties that were evaluated (N, CaMg, and Mn).

The study demonstrated that soil management can modulate FW in banana. However, the high plant mortality rates also indicate how serious the disease is. The rapid development of the disease clearly hampers the cultivation of Gros Michel banana as a perennial crop in Foc infested soils. An alternative scenario would be annual cropping or for a maximum of two crop cycles. Otherwise, replanting every 4–5 years can be considered for having an acceptable production under a high soil infestation with Foc. Implementing these alternatives requires a thorough cost/benefit analysis. Moreover, in a natural processes of plant inoculation (without the artificial forced inoculation in this experiment), a longer period to achieve the high disease mortality could be expected.

Nitrogen fertilization is an important cause for a lower soil pH in banana plantations. Particularly, the applied N ammonia sources in the experiments could decrease soil pH. This N source is linked with a higher incidence of fungal diseases in crops [58]. Soil management has been identified as an option to suppress FW in banana [59]. The results show that it can slow down the impact of FW, but that soil management is still unable to control the disease.

The significant effect of pH-low treatment can be associated to a higher Mn availability in these treatments. Soils in both experiments have high natural Mn concentrations. Natural Mn can be solubilized in pH-low, even in Mn-low treatments. Then the pH-Mn interaction can be partially causing plant mortality in the pH-low treatments. This interaction was more evident in the Exp-west with the lower soil fertility. The higher soil fertility in Exp-east could hide the effect of the soil properties (except to pH and N) and their interactions on the plant mortality. Moreover, the existence of the interactions of the soil properties in the expression of FW can imply a more complex scenario to use soil management to control or alleviate FW in banana.

The field experimentation brought along a high complexity to control the conditions in each experiment. The evaluated effect of soil management on FW expression was an indirect effect of the studied treatment. The disease expression in the plants was measured, but no data on the pathogen inoculum density in the soil were collected. Moreover, FW expression was certainly ruled by the artificial inoculation with Foc Race 1. Although the potential direct effect of soil properties on the Foc population in the soil was not measured, it would be hidden to a certain extent by the Foc inoculation. Future studies should include the measurement of the Foc population before the plant inoculation and monitor the concentrations during the experiment to disentangle the direct and indirect effects of the treatments.

Although inoculation took place twice before the rapid increase in plant mortality in the third cycle, it appears that the level of infestation was not critical to induce the disease in the 1st and 2nd crop cycle. Time appears to play an important role in the accumulative effect of the fungus in the soil and in the plant. This artificial inoculation obviously differs from natural plant infestation of the production system. However, the consistency of the difference in the plant mortality according to soil pH, even in the 3rd and 4th crop cycle, shows the importance of this soil property in FW development. In addition, the pathogen aggressiveness and dissemination could be influenced by the interaction of Foc with other biotic factors, such as nematodes [60] and the banana weevil [61], that may be influenced by soil management practices.

Experiments with diseases in perennial crops over a short period in, e.g., greenhouses may hide certain effects. Nevertheless, testing diseases in greenhouse experiments can give useful information about the relation of the soil and the crop disease, but it is typically concluded that it requires further evaluation at the field level [22]. In the current study, every crop cycle provided a different pattern of disease expression in relation to soil properties. The duration of experiments reported in the literature could be another reason for the inconsistent results in experiments with soil management [4].

The selection of representative locations is also important as illustrated by the differences between the two experiments were evident. This implies that only taking one subregion only tells part of the story, but also that the differences in ecological conditions can explain the inconsistent results in experiments with soil management and FW in bananas.

### 4.2. Soil Analysis for Tested Soil Properties

Some soil properties did not show significant differences in crop performance and FW. For example, the Ca concentration did not differ significantly between Ca-low and Ca-high. In addition, Ca and Mg concentrations dropped during the experiments. Similarly, despite the differences in N applications, the soil concentration did not differ significantly between N-high and N-low. This can be explained by the perhumid conditions in the region and the exceptionally high rainfall of 6900 mm during the 1st and 2nd crop cycle that may have led to considerable nutrient leaching [62]. This could have caused the general drop in soil pH and the lack of differences in soil conditions in both experiments in the 1st and 2nd crop cycle, as shown in the second soil analysis. Nevertheless, it is evident that soils from Exp-east maintained a higher soil fertility than soils in Exp-west during the whole experimental period, except for the N concentration. The binding of soil organic matter to the allophanes in the volcanic ash soils in Exp-west may explain this specific difference for N [46,47]. The influence of soil management was less apparent in the fertile soils in Exp-east as shown by the similarity in nutrients concentration in these soils despite the differences in treatments. The lower inherent soil fertility in Exp-west makes it more likely to exhibit differences as a result of soil management.

### 4.3. Leaf Nutrient Concentration According to Soil Management

The higher mortality in the 3rd and 4th cycle could be linked in one hand with a higher Foc density due the death plants and in the other hand by the nutritional status of the plants due as indicated by the differences in nutrient concentrations in leaf samples. This effect was cumulative, and it began in the 1st and 2nd crop cycle. Nutrient concentrations can be linked to the plant predisposition to Foc [16,17,19,63,64]. However, it is difficult to draw hard conclusions as the fungus will damage the vessels in the plant resulting in problems with nutrient transport. Although soil pH and N can influence plant mortality by FW, integrated nutrient management based on leaf analysis can be part of the strategy to reduce the predisposition of the crop to the disease.

### 4.4. Agronomic Performance According to Soil Management

Soil management influenced agronomic performance as expected [36] in the 1st and 2nd cycle. It appears that it is possible to maintain an acceptable crop production with a low impact of the disease at least during those crop cycles. Potentially, more intensive changes to soil management can delay the rapid increase in mortality. However, the agronomic performance of the “surviving plants” shows that production could be maintained in the first crop cycles after the inoculation. Although the infection appears to be devasting, gaining time to find alternative strategies through implementing practices, such as early detection and eradication of infested plants, replanting annual cropping, and crop rotation, may help to reduce yield losses considerably.

## 5. Conclusions

This study confirmed the potential role of soil management on FW expression in banana. The results of the field experiment were comparable to earlier results in greenhouse experiments with a very clear effect of soil pH. Effects were most significant until the 3rd crop cycle after which the aggregated effect became very serious with very high mortality rates. Soil pH appeared to be a key factor explaining differences in mortality rates. A higher soil pH can alleviate FW expression in the first two crop cycles after planting. However, the interactions between the various soil properties limit the development of specific soil management strategies for FW control. Moreover, differences in plant mortality appear to be relatively small and not warrant the investment of a large soil management program to control FW in bananas at the farm level. However, maintaining a higher soil pH and applying adequate N doses and Ca and Mg fertilizers can be included as part of the strategy, focusing on reducing plant mortality and maintaining the production in the short and probably the medium term. Otherwise, these practices can be integrated with other crop management techniques, such as the early detection and eradication of infested plants to reduce inoculum pressure, replanting, annual cropping, and crop rotation, to alleviate the impact of FW on the production. This kind of medium term field experimentation is essential for banana and other perennial crops as the effects may clearly differ over the crop cycles. This allows to evaluate the accumulated effect of the disease over time and to minimize the risk of imprecise conclusions induced by partial results obtained in short term studies. 

## Figures and Tables

**Table 1:** Treatments in the factorial experiment to study the effect of managing soil chemical properties on FW in Gros Michel banana in the Costa Rican banana region.

Soil Management		Application	Timing
General			
	Compost	1 kg per plant in each planting hole	At planting
	Startup fertilization	100 N, 18 K_2_O, 27 MgO, 53 CaO, 20 S, 5.8 Zn and 0.8 B (in kg ha^−1^ year^−1^)	During the first 7 weeks, divided over weekly applications.
	Basic nutritional package [36]	105 P_2_O_5_, 610 K_2_O, 4.0 CaO, 60 S, 9.2 Zn and 3.2 B (in kg ha^−1^ year^−1^)	After week 8, divided over 17 3-weekly applications per year
Treatments			
	pH	Low: 300 mL of 5% HCl solution and 303 g of CaO ^1^ per plant per application High: 400 g of Ca(OH)_2_ per plant per application	1 week before planting; 8 weeks after planting; between cycle 2 and 3
	N	Low: 232 kg N ha^−1^ year^−1^ with NH_4_NO_3_ and CaNO_3_ High: 605 kg ha^−1^ year^−1^ with NH_4_NO_3_ and CaNO_3_	Starting in week 8. Divided over 17 3-weekly applications per year
	CaMg ^2^	Low: - High: 315 kg CaO ha^−1^ year^−1^ with Ca_3_(BO_3_)_2_ and CaNO_3._ 150 kg MgO ha^−1^ year^−1^ with MgSO_4_	Starting in week 8. Divided over 17 3-weekly applications per year
	Mn	Low: - High: 32 kg Mn ha^−1^ year^−1^ with MnSO_4_	Starting in week 8. Divided over 17 3-weekly applications per year

^1^ CaO is applied to the low pH treatment to compensate for the Ca input in the high pH treatment. ^2^ Ca is also applied with the pH treatments and as part of the basic nutritional package as it comes with the borate application.

**Table 2:** Soil chemical properties in two experiments (Exp-west and Exp-east) from two locations (La Rita and 28 Millas respectively) of the Costa Rican banana region during the experimental period of four crop cycles. (*n* = 32 per soil chemical property in each region and crop cycle).

Treatment	Units	Soil Analysis		
		Start	Cycle 1–2	Cycle 3–4
Exp-west (La Rita)				
N-low	%	n.d	0.30	0.30
N-high	%	n.d	0.31	0.30
pH-low	-	5.5	5.4 ***	5.1 ***
pH-high	-	5.5	5.7	5.6
Ca-low	cmol (+) kg^−1^	4.60	4.18	4.06
Ca-high	cmol (+) kg^−1^	4.60	4.26	4.26
Mg-low	cmol (+) kg^−1^	2.90	1.41 **	1.84 ***
Mg-high	cmol (+) kg^−1^	2.90	1.67	2.35
Mn-low	mg kg^−1^	11	15 ***	16 ***
Mn-high	mg kg^−1^	11	18	23
Exp-east (28 Millas)				
N-low	%	n.d	0.16	0.15
N-high	%	n.d	0.17	0.14
pH-low	-	6.3	5.4 **	5.4 ***
pH-high	-	6.3	5.8	5.9
Ca-low	cmol (+) kg^−1^	22.2	21.9	22.6
Ca-high	cmol (+) kg^−1^	22.2	21.8	22.8
Mg-low	cmol (+) kg^−1^	10.9	9.65	9.83
Mg-high	cmol (+) kg^−1^	10.9	9.54	10.2
Mn-low	mg kg^−1^	88	90 ***	88
Mn-high	mg kg^−1^	88	133	87

** *p* < 0.010, *** *p* < 0.001; comparison between the low and the high level of each soil property.

**Table 3:** Leaf nutrient concentrations at the first and the second crop cycles of Gros Michel banana inoculated with Foc Race 1 in relation with two levels (high and low) of four soil chemical properties (pH, N, CaMg and Mn) in two experiments (Exp-west and Exp-east) from two locations (La Rita and 28 Millas respectively) in the Costa Rican banana region. (*n* = 32 per soil property per crop cycle and level of each soil chemical property).

Treatment	N	P	K	Ca	Mg	S	Fe	Cu	Zn	Mn	B
	% dry matter^–1^						mg Kg^–1^				
	Exp-west (La Rita), 1st cycle										
pH-low	2.93	0.21	4.32	0.60	0.28	0.19	67	9	17	172 *	8 *
pH-high	2.90	0.21	4.39	0.57	0.27	0.19	64	9	17	147	9
N-low	2.86	0.21	4.43	0.56 *	0.27	0.19 *	67	9	17	149 *	9
N-high	2.96	0.21	4.28	0.61	0.29	0.20	64	9	16	169	8
CaMg-low	2.92	0.21	4.34	0.58	0.27 *	0.19	67	9	17	165	8
CaMg-high	2.91	0.21	4.36	0.59	0.29	0.19	64	9	17	154	9
Mn-low	2.89	0.21	4.40	0.57	0.28	0.19	65	9	17	149 *	9
Mn-high	2.94	0.21	4.30	0.60	0.28	0.20	66	9	17	170	8
	Exp-west (La Rita), 2nd cycle										
pH-low	2.55	0.19	3.77	0.67	0.28	0.18	58	7	17	183 *	9 *
pH-high	2.54	0.19	3.82	0.75	0.26	0.18	57	7	17	163	11
N-low	2.50 *	0.20 *	3.86 *	0.66 *	0.27	0.19 *	59 *	7	18 *	155 *	10 *
N-high	2.59	0.18	3.73	0.76	0.27	0.18	56	7	17	190	9
CaMg-low	2.53	0.19	3.80	0.71	0.26 *	0.18	58	7	17	176	10
CaMg-high	2.56	0.19	3.79	0.71	0.28	0.18	57	7	17	170	10
Mn-low	2.53	0.19	3.83	0.71	0.27	0.18	58	7	17	157 *	10
Mn-high	2.56	0.19	3.76	0.72	0.27	0.18	58	7	17	189	10
	Exp-east (28 Millas), 1st cycle										
pH-low	2.42	0.19	3.70 *	0.66	0.28	0.17	58	7	17 *	408	12
pH-high	2.44	0.19	3.58	0.63	0.27	0.17	56	7	16	351	12
N-low	2.34 *	0.20 *	3.73 *	0.60 *	0.26 *	0.17	57	7	17	355	12
N-high	2.52	0.19	3.55	0.69	0.28	0.17	58	7	17	403	11
CaMg-low	2.44	0.19	3.74 *	0.65	0.27	0.17	58	7	17	378	12
CaMg-high	2.43	0.19	3.54	0.64	0.28	0.17	57	7	17	382	12
Mn-low	2.42	0.19	3.71 *	0.64	0.27	0.17	57	7	17	366 *	11
Mn-high	2.45	0.19	3.57	0.65	0.28	0.17	58	7	17	394	12
	Exp-east (28 Millas), 2nd cycle										
pH-low	2.50	0.19	3.27	0.81 *	0.36	0.18	66	8	17	298	10
pH-high	2.49	0.19	3.27	0.89	0.36	0.18	67	7	17	272	11
N-low	2.43 *	0.20 *	3.39 *	0.78 *	0.34 *	0.18	67	7	17	289	11
N-high	2.55	0.18	3.16	0.91	0.38	0.18	67	8	17	282	10
CaMg-low	2.50	0.19	3.34	0.85	0.36	0.18	67	8	17	294	11
CaMg-high	2.49	0.19	3.21	0.85	0.37	0.18	67	8	17	276	10
Mn-low	2.51	0.20 *	3.29	0.83 *	0.36	0.18	67	8	17	287	11
Mn-high	2.48	0.18	3.26	0.87	0.36	0.18	66	7	17	283	10

* *p* < 0.050; comparison between the low and the high level of each soil treatment.

**Table 4:** Agronomic performance in the 1st and 2nd of Gros Michel banana inoculated with Foc Race 1 in two experiments (Exp-west and Exp-east) from two locations (La Rita and 28 Millas respectively) of the Costa Rican banana region. (*n* = 64 in both experiments).

Variable	Exp-West La Rita		Exp-East 28 Millas	
	1st Cycle	2nd Cycle	1st Cycle	2nd Cycle
Plant height (cm)	337	369	300	355
Pseudostem circumference (cm)	75	83	64	79
Bunch weight (kg)	25.2	29	19.3	24.8
Clusters bunch^–1^	9.2	10.3	8.5	8.9
Harvested fruit ^§^ (ton ha^–1^)	39.9	41.3	30.6	31.8

^§^: 1600 plant ha^–1^ × (100% – % mortality) × average bunch weight in each cycle.

## Data Availability

Data set supporting this publication is available in https://osf.io/2jyse.
